# Diacetonitrile­tetra­kis{μ_2_-3-anilinocarbonyl-1-[(5-chloro-2-oxidophen­yl)diazen­yl]-2-naphtholato}tetra­aqua­diiron(III)disodium(I) dihydrate

**DOI:** 10.1107/S1600536807066895

**Published:** 2007-12-21

**Authors:** Sato Yohei, Uta Kazuya, Jin Mizuguchi

**Affiliations:** a Department of Applied Physics, Graduate School of Engineering, Yokohama National University, 79-5 Tokiwadai, Hodogaya-ku, 240-8501 Yokohama, Japan

## Abstract

The title compound, [Fe_2_Na_2_(C_23_H_14_ClN_3_O_3_)_4_(C_2_H_3_N)_2_(H_2_O)_4_]·2H_2_O, is a hydrated Fe–azo complex dimer that is used as a charge-control agent in electrophotography. The mol­ecule is a centrosymmetric dimer with two octa­hedral Fe^III^ units linked by two bridging five-coordinate Na^I^ cations. Each Fe^III^ atom is chelated by the N and two O atoms from two 3-anilinocarbonyl-1-[(5-chloro-2-oxidophen­yl)diazen­yl]-2-naph­tholate ligands. The Na^+^ cation is coordinated by a carbonyl O atom from the two ligands of each octa­hedral Fe^III^ unit, two water mol­ecules and the N atom of an acetonitrile mol­ecule. Two solvent water mol­ecules complete the structure. In the crystal structure, the dimeric mol­ecules are bridged by a pair of discrete inter­molecular O—H⋯O hydrogen bonds, one of which involves a sodium-bound water mol­ecule and a hydrate water, and the other a 5-chloro­phenolate O atom and a water molecule to form an extended chain along *b*.

## Related literature

For general background to charge-control agents, see Tanaka (1995 ▶); and for the preparation of the title compound, see Yasumatsu *et al.* (2006 ▶). For related structures, see: Mizuguchi, Sato, Uta & Sato (2007 ▶); Mizuguchi *et al.* (2007*a* ▶,*b* ▶); Mizuguchi, Uta & Sato (2007 ▶).
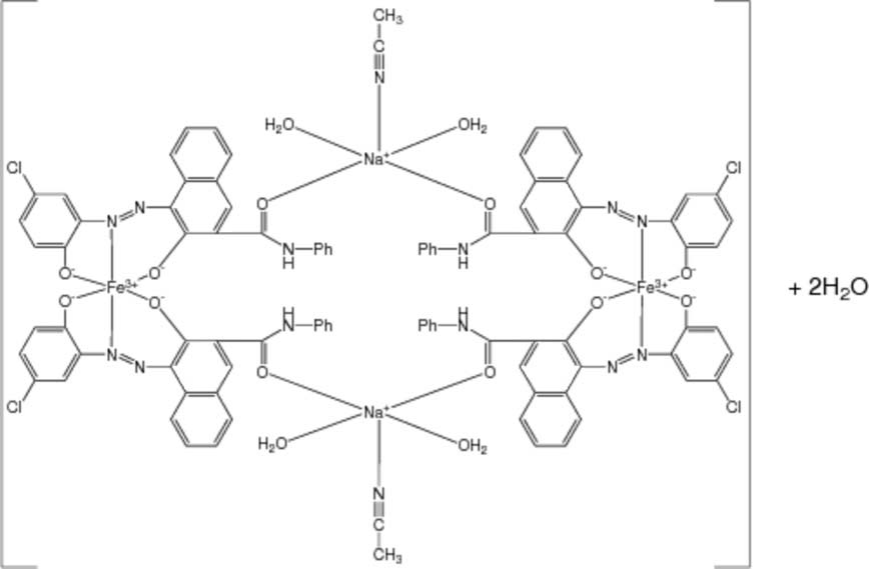


## Experimental

### 

#### Crystal data

[Fe_2_Na_2_(C_23_H_14_ClN_3_O_3_)_4_(C_2_H_3_N)_2_(H_2_O)_4_]·2H_2_O
*M* *_r_* = 2011.21
Triclinic, 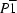

*a* = 11.4416 (5) Å
*b* = 14.1161 (7) Å
*c* = 15.0105 (7) Å
α = 72.396 (1)°
β = 76.2850 (9)°
γ = 83.015 (1)°
*V* = 2241.67 (18) Å^3^
*Z* = 1
Mo *K*α radiation
μ = 0.53 mm^−1^
*T* = 93.1 K
0.30 × 0.09 × 0.05 mm

#### Data collection

Rigaku R-AXIS RAPID diffractometer
Absorption correction: multi-scan (*ABSCOR*; Higashi, 1995 ▶) *T*_min_ = 0.793, *T*_max_ = 0.976
40907 measured reflections
8133 independent reflections
4163 reflections with *I* > 2σ(*I*)
*R*_int_ = 0.125

#### Refinement

*R*[*F* ^2^ > 2σ(*F* ^2^)] = 0.086
*wR*(*F* ^2^) = 0.264
*S* = 0.97
8133 reflections
607 parameters
H-atom parameters constrained
Δρ_max_ = 1.17 e Å^−3^
Δρ_min_ = −1.00 e Å^−3^

### 

Data collection: *PROCESS-AUTO* (Rigaku, 1998 ▶); cell refinement: *PROCESS-AUTO*; data reduction: *CrystalStructure* (Rigaku/MSC, 2006 ▶); program(s) used to solve structure: *SIR2004* (Burla *et al.*, 2005 ▶); program(s) used to refine structure: *SHELXL97* (Sheldrick, 1997 ▶); molecular graphics: *ORTEPIII* (Burnett & Johnson, 1996 ▶); software used to prepare material for publication: *CrystalStructure*.

## Supplementary Material

Crystal structure: contains datablocks global, I. DOI: 10.1107/S1600536807066895/sj2453sup1.cif

Structure factors: contains datablocks I. DOI: 10.1107/S1600536807066895/sj2453Isup2.hkl

Additional supplementary materials:  crystallographic information; 3D view; checkCIF report

## Figures and Tables

**Table:** 

Fe1—O1	1.970 (4)
Fe1—O2	1.944 (3)
Fe1—O3	1.986 (3)
Fe1—O4	1.976 (4)
Fe1—N1	2.127 (4)
Fe1—N4	2.161 (4)
Na1—O5^i^	2.222 (4)
Na1—O6	2.238 (5)
Na1—O7	2.508 (7)
Na1—O8	2.329 (6)
Na1—N7	2.381 (9)

**Table:** 

O1—Fe1—O2	90.26 (17)
O1—Fe1—O3	85.60 (17)
O1—Fe1—O4	160.14 (15)
O1—Fe1—N1	81.72 (18)
O1—Fe1—N4	111.90 (19)
O2—Fe1—O3	155.71 (17)
O2—Fe1—O4	96.86 (18)
O2—Fe1—N1	100.35 (16)
O2—Fe1—N4	79.14 (16)
O3—Fe1—O4	95.02 (17)
O3—Fe1—N1	102.70 (16)
O3—Fe1—N4	80.26 (17)
O4—Fe1—N1	78.78 (18)
O4—Fe1—N4	87.69 (18)
N1—Fe1—N4	166.3 (2)
O5^i^—Na1—O6	97.7 (2)
O5^i^—Na1—O7	88.35 (19)
O5^i^—Na1—O8	108.0 (2)
O5^i^—Na1—N7	149.5 (3)
O6—Na1—O7	92.9 (2)
O6—Na1—O8	101.3 (2)
O6—Na1—N7	107.6 (2)
O7—Na1—O8	156.4 (2)
O7—Na1—N7	73.9 (2)
O8—Na1—N7	83.8 (2)

Symmetry codes: (i) 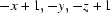; (ii) 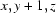; (iii) 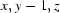.

**Table 2:** Hydrogen-bond geometry (Å, °)

*D*—H⋯*A*	*D*—H	H⋯*A*	*D*⋯*A*	*D*—H⋯*A*
N3—H3N⋯O1	0.86	1.94	2.644 (7)	138
N6—H6N⋯O3	0.86	1.97	2.664 (6)	138
O8—H⋯O9	–	–	2.730 (7)	–
O9—H⋯O4^iii^	–	–	2.710 (6)	–

Symmetry code: (iii) 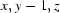.

**Fig. 1.**
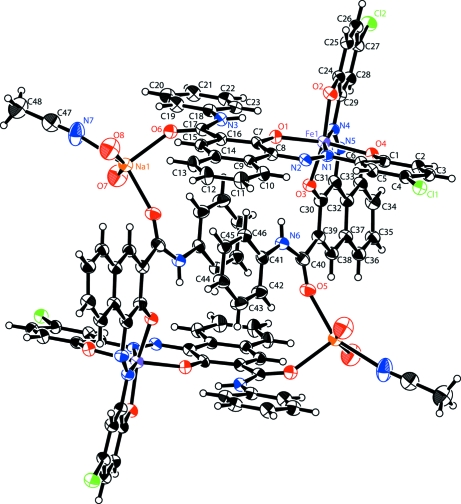
A view of the molecular structure of (I), showing 30% displacement ellipsoids.

**Fig. 2.**
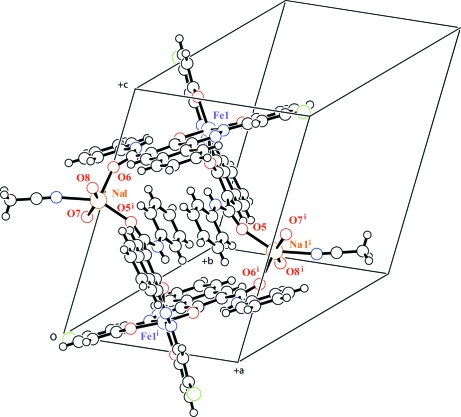
The centrosymmetric complex dimer. The two Fe-azo complexes are bridged by Na1 and Na1i cations through Na1—O5i and Na1—O6, and Na1i—O5 and Na1i—O6i bonds, respectively [symmetry code(i): 1 - x, -y, 1 - z].

**Fig. 3.**
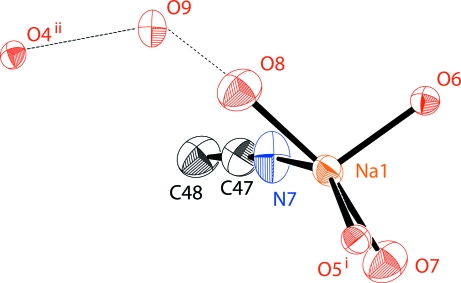
shows two distinct intermolecular O···O interactions, dashed lines, that link two azo-Fe complex dimers: O8···O9 and O9···O4ii Hydrogen atoms on O8 and O9 could not be located in difference electron density maps [symmetry codes (i): -x + 1, -y, -z + 1 and (ii): x, -1 + y, z].

## supplementary crystallographic information

### Comment

The title compound, I, is a hydrated Fe-azo complex dimer that is used as a charge-control agent (CCA) of the negative type in electrophotography (Tanaka, 1995). The purpose of the investigation has been set out in our previous paper (Mizuguchi, Sato, Uta & Sato, 2007). We have previously reported the structure of a methanol-solvated Fe-azo complex with an ammonium cation (Mizuguchi *et al.*, 2007*a*) and its acetone solvate (Mizuguchi, Uta & Sato, 2007). together with the structure of an acetone-solvated Fe-azo complex with a sodium cation, in place of the ammonium (Mizuguchi *et al.*, 2007*b*). In this molecule, the sodium cation is found to bridge three different Fe-azo complexes through the Na—O bonds between the sodium cation and the carbonyl O atoms of the Fe-azo complex. The present paper reports a related hydrated Fe-azo complex dimer bridged by two sodium cations.

Fig. 1 shows the asymmetric unit of the complex with the complete centrosymmetric dimer molecule shown in Fig. 2. The dimer comprises two octahedral Fe^III^ units linked by two bridging five-coordinate Na^I^ cations. Each Fe^III^ atom is chelated by the N and two O atoms from two 3-anilinocarbonyl-1-[(5-chloro-2-oxidophenyl)diazenyl]-2-naphtholate ligands with the naphtholate and chloro-2-oxidophenyl O atoms mutually *cis*. In each octahedral Fe^III^ unit, there are two intramolecular N3—H3N···O1 and N6—H6N···O3 hydrogen bonds that effect the conformation of the molecule (Table 2). The five-coordinate Na cation binds to N7 of the acetonitrile molecule, the O7 and O8 atoms of the two coordinated water molecules, and to the O6 and O5^i^ carbonyl O atoms from of each octahedral Fe^III^ unit [symmetry code (i): 1 - *x*, -*y*, 1 - *z*]). There are also two hydrated water molecules O9 and O9^ii^ [symmetry code (ii): *x*, *y* - 1,*z*].

In the crystal structure the dimeric molecules are bridged by a pair of discrete intermolecular O—H···O hydrogen bonds as shown in Fig. 3. One of these involves a sodium bound water molecule and a hydrate water, and the other a 5-chlorphenolate O atom and a hydrate water to form an extended chain along *b*. The atoms involved are: O8—(H)···O9 and O9—(H)···O4^ii^ [symmetry code: (ii) *x*, -1 + *y*, *z*] with the H atoms in parenthesis not located. The H atoms on the O7, O8 and O9 atoms of the water molecules could not be found in difference density maps. However the bond distances O8—O9, 2.730 (7) and O9—O4^ii^, 2.710 (6) Å [symmetry code: (ii) *x*, -1 + *y*, *z*] strongly suggest the presence of classical hydrogen bonds.

### Experimental

Compound I was prepared according to the previously reported method (Yasumatsu *et al.*, 2006). Single crystals of (I) were recrystallized from an acetonitrile solution as blocks over a 48 h period.

### Refinement

All H atoms were placed in geometrically idealized positions and constrained to ride on their parent atoms, with C—H = 0.93 except for C—H in methyl groups (0.96) and N—H = 0.86 A, and *U*_iso_ (H) = 1.2 *U*_eq_ (parent atom). Six H atoms on the three water molecules (O7, O8 and O9) were not located in difference electron density maps.

R-merge for the reflection data was 12.5%. This indicates poor crystal quality resulting in a rather high value of the *R* factor.

### Crystal data

**Table:**

[Fe_2_Na_2_(C_23_H_14_ClN_3_O_3_)_4_(C_2_H_3_N)_2_(H_2_O)_4_]·2H_2_O	*Z* = 1
*M**_r_* = 2011.21	*F*_000_ = 1022.0
Triclinic, *P*1	*D*_x_ = 1.481 Mg m^−^^3^
Hall symbol: -P 1	Mo *K*α radiation λ = 0.71075 Å
*a* = 11.4416 (5) Å	Cell parameters from 18467 reflections
*b* = 14.1161 (7) Å	θ = 3.0–25.3º
*c* = 15.0105 (7) Å	µ = 0.53 mm^−^^1^
α = 72.396 (1)º	*T* = 93.1 K
β = 76.2850 (9)º	Block, black
γ = 83.015 (1)º	0.30 × 0.09 × 0.05 mm
*V* = 2241.67 (18) Å^3^	

### Data collection

**Table:**

Rigaku R-AXIS RAPID diffractometer	4163 reflections with *I* > 2σ(*I*)
Detector resolution: 10.00 pixels mm^-1^	*R*_int_ = 0.125
ω scans	θ_max_ = 25.4º
Absorption correction: multi-scan(ABSCOR; Higashi, 1995)	*h* = −13→13
*T*_min_ = 0.793, *T*_max_ = 0.976	*k* = −16→16
40907 measured reflections	*l* = −18→18
8133 independent reflections	

### Refinement

**Table:**

Refinement on *F*^2^	H-atom parameters constrained
*R*[*F*^2^ > 2σ(*F*^2^)] = 0.086	*w* = 1/[σ^2^(*F*_o_^2^) + (0.1531*P*)^2^] where *P* = (*F*_o_^2^ + 2*F*_c_^2^)/3
*wR*(*F*^2^) = 0.264	(Δ/σ)_max_ < 0.001
*S* = 0.97	Δρ_max_ = 1.17 e Å^−^^3^
8133 reflections	Δρ_min_ = −1.00 e Å^−^^3^
607 parameters	Extinction correction: none

### Special details

**Table:**

Geometry. All e.s.d.'s (except the e.s.d. in the dihedral angle between two l.s. planes) are estimated using the full covariance matrix. The cell e.s.d.'s are taken into account individually in the estimation of e.s.d.'s in distances, angles and torsion angles; correlations between e.s.d.'s in cell parameters are only used when they are defined by crystal symmetry. An approximate (isotropic) treatment of cell e.s.d.'s is used for estimating e.s.d.'s involving l.s. planes.
Refinement. Refinement of *F*^2^ against ALL reflections. The weighted *R*-factor *wR* and goodness of fit *S* are based on *F*^2^, conventional *R*-factors *R* are based on *F*, with *F* set to zero for negative *F*^2^. The threshold expression of *F*^2^ > σ(*F*^2^) is used only for calculating *R*-factors(gt) *etc*. and is not relevant to the choice of reflections for refinement. *R*-factors based on *F*^2^ are statistically about twice as large as those based on *F*, and *R*- factors based on ALL data will be even larger.

### Fractional atomic coordinates and isotropic or equivalent isotropic displacement parameters (Å^2^)

**Table:**

	*x*	*y*	*z*	*U*_iso_*/*U*_eq_	
Fe1	0.35889 (7)	0.15898 (6)	0.81868 (6)	0.0419 (3)	
Cl1	0.89755 (14)	0.08974 (12)	0.97345 (11)	0.0493 (4)	
Cl2	−0.13236 (15)	0.46998 (12)	0.94460 (12)	0.0570 (4)	
Na1	0.1669 (2)	−0.2788 (2)	0.66698 (19)	0.0631 (7)	
O1	0.3035 (3)	0.0314 (2)	0.8208 (2)	0.0431 (9)	
O2	0.2505 (3)	0.1532 (2)	0.9412 (2)	0.0455 (10)	
O3	0.4037 (3)	0.1844 (2)	0.6776 (2)	0.0441 (10)	
O4	0.4653 (3)	0.2573 (2)	0.8224 (2)	0.0435 (10)	
O5	0.6633 (4)	0.2145 (3)	0.4266 (3)	0.0565 (11)	
O6	0.1263 (3)	−0.1752 (3)	0.7588 (2)	0.0506 (11)	
O7	0.0555 (5)	−0.1671 (4)	0.5459 (4)	0.1103 (15)	
O8	0.2170 (5)	−0.4270 (4)	0.7749 (4)	0.1103 (15)	
O9	0.3764 (4)	−0.5671 (3)	0.8564 (3)	0.0660 (13)	
N1	0.5057 (4)	0.0648 (3)	0.8650 (3)	0.0397 (11)	
N2	0.5346 (4)	−0.0289 (3)	0.8773 (3)	0.0398 (11)	
N3	0.1124 (4)	−0.0205 (3)	0.7800 (3)	0.0454 (12)	
N4	0.2318 (4)	0.2816 (3)	0.7769 (3)	0.0413 (11)	
N5	0.2329 (4)	0.3526 (3)	0.6989 (3)	0.0430 (12)	
N6	0.5554 (4)	0.1015 (3)	0.5544 (3)	0.0449 (12)	
N7	−0.0136 (6)	−0.3582 (5)	0.6870 (6)	0.101 (2)	
C1	0.5628 (5)	0.2210 (4)	0.8598 (4)	0.0422 (14)	
C2	0.6393 (5)	0.2825 (4)	0.8750 (4)	0.0448 (15)	
C3	0.7400 (5)	0.2409 (4)	0.9114 (4)	0.0473 (15)	
C4	0.7679 (5)	0.1382 (4)	0.9319 (4)	0.0451 (14)	
C5	0.6932 (5)	0.0759 (4)	0.9196 (4)	0.0428 (14)	
C6	0.5913 (5)	0.1184 (4)	0.8821 (4)	0.0436 (14)	
C7	0.3479 (5)	−0.0602 (4)	0.8343 (4)	0.0392 (13)	
C8	0.4605 (5)	−0.0899 (4)	0.8615 (4)	0.0386 (13)	
C9	0.5082 (5)	−0.1914 (4)	0.8752 (4)	0.0402 (14)	
C10	0.6220 (5)	−0.2231 (4)	0.8991 (4)	0.0486 (15)	
C11	0.6632 (6)	−0.3209 (4)	0.9089 (5)	0.0593 (18)	
C12	0.5984 (6)	−0.3902 (4)	0.8959 (5)	0.0592 (18)	
C13	0.4881 (6)	−0.3611 (4)	0.8720 (5)	0.0544 (17)	
C14	0.4414 (5)	−0.2609 (4)	0.8604 (4)	0.0454 (15)	
C15	0.3295 (5)	−0.2294 (4)	0.8330 (4)	0.0450 (14)	
C16	0.2828 (5)	−0.1335 (4)	0.8186 (4)	0.0398 (13)	
C17	0.1688 (5)	−0.1119 (4)	0.7845 (4)	0.0424 (14)	
C18	0.0062 (5)	0.0210 (4)	0.7457 (4)	0.0452 (15)	
C19	−0.0736 (6)	−0.0332 (5)	0.7267 (4)	0.0573 (17)	
C20	−0.1751 (6)	0.0128 (5)	0.6939 (4)	0.0605 (18)	
C21	−0.1973 (6)	0.1163 (5)	0.6778 (4)	0.0573 (18)	
C22	−0.1186 (5)	0.1703 (5)	0.6963 (4)	0.0530 (17)	
C23	−0.0168 (5)	0.1234 (5)	0.7317 (4)	0.0502 (16)	
C24	0.1631 (5)	0.2232 (4)	0.9440 (4)	0.0415 (14)	
C25	0.0855 (5)	0.2317 (4)	1.0296 (4)	0.0434 (14)	
C26	−0.0026 (5)	0.3072 (4)	1.0291 (4)	0.0471 (15)	
C27	−0.0182 (5)	0.3765 (4)	0.9418 (4)	0.0444 (14)	
C28	0.0548 (5)	0.3704 (4)	0.8564 (4)	0.0468 (15)	
C29	0.1455 (5)	0.2944 (4)	0.8573 (4)	0.0421 (14)	
C30	0.4013 (5)	0.2678 (4)	0.6089 (4)	0.0452 (15)	
C31	0.3192 (5)	0.3486 (4)	0.6182 (4)	0.0456 (15)	
C32	0.3197 (5)	0.4395 (4)	0.5399 (4)	0.0437 (14)	
C33	0.2371 (5)	0.5215 (4)	0.5450 (4)	0.0484 (15)	
C34	0.2456 (6)	0.6069 (4)	0.4700 (4)	0.0541 (17)	
C35	0.3359 (6)	0.6164 (4)	0.3864 (4)	0.0548 (17)	
C36	0.4139 (6)	0.5365 (4)	0.3802 (4)	0.0510 (16)	
C37	0.4090 (5)	0.4471 (4)	0.4548 (4)	0.0442 (14)	
C38	0.4904 (5)	0.3653 (4)	0.4471 (4)	0.0476 (15)	
C39	0.4874 (5)	0.2775 (4)	0.5180 (4)	0.0452 (15)	
C40	0.5767 (6)	0.1949 (5)	0.4971 (4)	0.0505 (16)	
C41	0.6173 (5)	0.0108 (4)	0.5470 (4)	0.0435 (14)	
C42	0.7141 (5)	0.0022 (5)	0.4734 (4)	0.0564 (17)	
C43	0.7690 (6)	−0.0910 (5)	0.4735 (5)	0.068 (2)	
C44	0.7300 (6)	−0.1763 (5)	0.5459 (4)	0.0577 (17)	
C45	0.6337 (5)	−0.1664 (5)	0.6187 (4)	0.0523 (16)	
C46	0.5787 (5)	−0.0738 (4)	0.6184 (4)	0.0462 (15)	
C47	−0.0920 (8)	−0.4004 (6)	0.6882 (6)	0.081 (2)	
C48	−0.1940 (8)	−0.4558 (7)	0.6902 (6)	0.103 (3)	
H2	0.6224	0.3509	0.8605	0.054*	
H3	0.7899	0.2816	0.9224	0.057*	
H3N	0.1456	0.0176	0.8008	0.054*	
H5	0.7097	0.0074	0.9356	0.051*	
H6N	0.4958	0.0970	0.6024	0.054*	
H10	0.6683	−0.1784	0.9082	0.058*	
H11	0.7379	−0.3410	0.9248	0.071*	
H12	0.6288	−0.4557	0.9033	0.071*	
H13	0.4439	−0.4072	0.8634	0.066*	
H15	0.2858	−0.2758	0.8242	0.053*	
H19	−0.0590	−0.1018	0.7363	0.068*	
H20	−0.2287	−0.0245	0.6825	0.073*	
H21	−0.2649	0.1478	0.6548	0.069*	
H22	−0.1329	0.2388	0.6852	0.064*	
H23	0.0350	0.1602	0.7457	0.060*	
H25	0.0940	0.1857	1.0872	0.052*	
H26	−0.0521	0.3128	1.0863	0.057*	
H28	0.0441	0.4159	0.7991	0.056*	
H33	0.1765	0.5177	0.5995	0.058*	
H34	0.1901	0.6600	0.4747	0.065*	
H35	0.3421	0.6754	0.3369	0.066*	
H36	0.4726	0.5412	0.3246	0.061*	
H38	0.5489	0.3713	0.3911	0.057*	
H42	0.7418	0.0583	0.4246	0.068*	
H43	0.8335	−0.0967	0.4239	0.082*	
H44	0.7678	−0.2384	0.5453	0.069*	
H45	0.6060	−0.2222	0.6679	0.063*	
H46	0.5138	−0.0684	0.6677	0.055*	
H48A	−0.1883	−0.4635	0.6279	0.123*	
H48B	−0.2682	−0.4198	0.7081	0.123*	
H48C	−0.1919	−0.5202	0.7358	0.123*	

### Atomic displacement parameters (Å^2^)

**Table:**

	*U*^11^	*U*^22^	*U*^33^	*U*^12^	*U*^13^	*U*^23^
Fe1	0.0437 (5)	0.0388 (5)	0.0420 (5)	0.0055 (3)	−0.0130 (4)	−0.0097 (3)
Cl1	0.0459 (8)	0.0517 (9)	0.0505 (9)	0.0036 (7)	−0.0165 (7)	−0.0122 (7)
Cl2	0.0557 (10)	0.0513 (10)	0.0614 (11)	0.0143 (7)	−0.0124 (8)	−0.0188 (8)
Na1	0.0676 (17)	0.0632 (18)	0.0588 (17)	−0.0126 (14)	0.0001 (13)	−0.0248 (13)
O1	0.044 (2)	0.040 (2)	0.046 (2)	0.0014 (18)	−0.0145 (19)	−0.0111 (18)
O2	0.049 (2)	0.041 (2)	0.047 (2)	0.0134 (19)	−0.0170 (19)	−0.0129 (19)
O3	0.049 (2)	0.040 (2)	0.041 (2)	0.0019 (18)	−0.0088 (19)	−0.0106 (18)
O4	0.044 (2)	0.040 (2)	0.049 (2)	0.0070 (18)	−0.0202 (19)	−0.0115 (18)
O5	0.057 (2)	0.054 (2)	0.052 (2)	0.006 (2)	−0.002 (2)	−0.016 (2)
O6	0.049 (2)	0.053 (2)	0.051 (2)	−0.001 (2)	−0.013 (2)	−0.014 (2)
O7	0.114 (3)	0.112 (3)	0.107 (3)	−0.011 (2)	−0.047 (3)	−0.014 (2)
O8	0.114 (3)	0.112 (3)	0.107 (3)	−0.011 (2)	−0.047 (3)	−0.014 (2)
O9	0.067 (3)	0.050 (2)	0.084 (3)	−0.001 (2)	−0.019 (2)	−0.022 (2)
N1	0.038 (2)	0.040 (2)	0.039 (2)	0.001 (2)	−0.007 (2)	−0.011 (2)
N2	0.050 (3)	0.033 (2)	0.034 (2)	0.005 (2)	−0.011 (2)	−0.009 (2)
N3	0.046 (2)	0.046 (3)	0.047 (3)	0.003 (2)	−0.015 (2)	−0.016 (2)
N4	0.042 (2)	0.040 (2)	0.041 (2)	0.001 (2)	−0.009 (2)	−0.012 (2)
N5	0.049 (3)	0.040 (2)	0.033 (2)	0.003 (2)	−0.010 (2)	−0.002 (2)
N6	0.050 (3)	0.039 (3)	0.041 (2)	0.003 (2)	−0.008 (2)	−0.009 (2)
N7	0.073 (4)	0.088 (5)	0.164 (8)	0.003 (4)	−0.057 (5)	−0.047 (5)
C1	0.042 (3)	0.047 (3)	0.037 (3)	0.003 (2)	−0.010 (2)	−0.012 (2)
C2	0.048 (3)	0.037 (3)	0.051 (3)	0.010 (2)	−0.022 (3)	−0.010 (2)
C3	0.046 (3)	0.044 (3)	0.052 (4)	−0.002 (2)	−0.005 (3)	−0.017 (2)
C4	0.045 (3)	0.046 (3)	0.047 (3)	−0.001 (2)	−0.014 (2)	−0.014 (2)
C5	0.048 (3)	0.041 (3)	0.042 (3)	0.007 (2)	−0.018 (2)	−0.013 (2)
C6	0.050 (3)	0.038 (3)	0.042 (3)	0.001 (2)	−0.016 (2)	−0.008 (2)
C7	0.046 (3)	0.034 (3)	0.041 (3)	0.005 (2)	−0.011 (2)	−0.018 (2)
C8	0.038 (3)	0.038 (3)	0.039 (3)	0.010 (2)	−0.016 (2)	−0.009 (2)
C9	0.044 (3)	0.038 (3)	0.039 (3)	0.007 (2)	−0.010 (2)	−0.013 (2)
C10	0.057 (3)	0.040 (3)	0.052 (3)	0.003 (3)	−0.020 (3)	−0.012 (2)
C11	0.052 (4)	0.050 (4)	0.080 (5)	0.016 (3)	−0.029 (3)	−0.021 (3)
C12	0.060 (4)	0.038 (3)	0.089 (5)	0.017 (3)	−0.034 (3)	−0.024 (3)
C13	0.059 (4)	0.039 (3)	0.072 (4)	−0.001 (3)	−0.019 (3)	−0.022 (3)
C14	0.050 (3)	0.038 (3)	0.048 (3)	0.003 (2)	−0.010 (2)	−0.015 (2)
C15	0.043 (3)	0.044 (3)	0.049 (3)	−0.001 (2)	−0.010 (2)	−0.015 (2)
C16	0.040 (3)	0.043 (3)	0.039 (3)	0.003 (2)	−0.017 (2)	−0.010 (2)
C17	0.047 (3)	0.037 (3)	0.041 (3)	0.005 (2)	−0.011 (2)	−0.010 (2)
C18	0.042 (3)	0.054 (4)	0.040 (3)	0.005 (3)	−0.013 (2)	−0.014 (2)
C19	0.055 (4)	0.060 (4)	0.059 (4)	0.014 (3)	−0.024 (3)	−0.018 (3)
C20	0.058 (4)	0.070 (5)	0.055 (4)	0.003 (3)	−0.019 (3)	−0.017 (3)
C21	0.055 (4)	0.061 (4)	0.060 (4)	0.015 (3)	−0.026 (3)	−0.019 (3)
C22	0.049 (3)	0.059 (4)	0.048 (4)	0.019 (3)	−0.019 (3)	−0.013 (3)
C23	0.049 (3)	0.057 (4)	0.043 (3)	0.005 (3)	−0.007 (2)	−0.016 (3)
C24	0.047 (3)	0.037 (3)	0.040 (3)	−0.001 (2)	−0.011 (2)	−0.009 (2)
C25	0.048 (3)	0.039 (3)	0.042 (3)	0.002 (2)	−0.015 (2)	−0.006 (2)
C26	0.046 (3)	0.049 (3)	0.048 (3)	0.003 (3)	−0.012 (2)	−0.017 (3)
C27	0.046 (3)	0.038 (3)	0.051 (3)	0.015 (2)	−0.016 (2)	−0.016 (2)
C28	0.053 (3)	0.039 (3)	0.047 (3)	0.005 (2)	−0.013 (3)	−0.012 (2)
C29	0.037 (3)	0.041 (3)	0.048 (3)	0.004 (2)	−0.014 (2)	−0.011 (2)
C30	0.053 (3)	0.041 (3)	0.037 (3)	−0.003 (2)	−0.012 (2)	−0.003 (2)
C31	0.058 (4)	0.034 (3)	0.046 (3)	0.008 (2)	−0.018 (3)	−0.013 (2)
C32	0.050 (3)	0.042 (3)	0.037 (3)	0.002 (2)	−0.012 (2)	−0.007 (2)
C33	0.055 (3)	0.045 (3)	0.046 (3)	0.006 (3)	−0.013 (3)	−0.014 (2)
C34	0.061 (4)	0.040 (3)	0.058 (4)	0.011 (3)	−0.019 (3)	−0.009 (3)
C35	0.073 (4)	0.045 (4)	0.041 (3)	−0.003 (3)	−0.009 (3)	−0.006 (2)
C36	0.062 (4)	0.047 (4)	0.043 (3)	−0.003 (3)	−0.011 (3)	−0.011 (2)
C37	0.051 (3)	0.043 (3)	0.037 (3)	0.006 (2)	−0.013 (2)	−0.010 (2)
C38	0.057 (4)	0.045 (3)	0.039 (3)	−0.004 (3)	−0.008 (3)	−0.010 (2)
C39	0.043 (3)	0.047 (3)	0.047 (3)	0.006 (2)	−0.014 (2)	−0.015 (2)
C40	0.056 (4)	0.054 (4)	0.044 (3)	0.004 (3)	−0.015 (3)	−0.015 (3)
C41	0.050 (3)	0.041 (3)	0.043 (3)	0.002 (2)	−0.015 (2)	−0.014 (2)
C42	0.060 (4)	0.046 (4)	0.051 (4)	0.000 (3)	0.000 (3)	−0.006 (3)
C43	0.070 (4)	0.052 (4)	0.064 (4)	−0.001 (3)	0.010 (3)	−0.008 (3)
C44	0.064 (4)	0.050 (4)	0.052 (4)	0.013 (3)	−0.006 (3)	−0.015 (3)
C45	0.054 (4)	0.048 (4)	0.051 (4)	−0.004 (3)	−0.011 (3)	−0.010 (3)
C46	0.047 (3)	0.047 (3)	0.041 (3)	0.003 (2)	−0.010 (2)	−0.009 (2)
C47	0.069 (5)	0.086 (6)	0.089 (6)	0.012 (4)	−0.027 (4)	−0.024 (4)
C48	0.097 (7)	0.106 (7)	0.105 (7)	−0.010 (5)	−0.039 (6)	−0.015 (5)

### Geometric parameters (Å, °)

**Table:**

Fe1—O1	1.970 (4)	C15—C16	1.366 (8)
Fe1—O2	1.944 (3)	C15—H15	0.930
Fe1—O3	1.986 (3)	C16—C17	1.477 (8)
Fe1—O4	1.976 (4)	C18—C19	1.387 (11)
Fe1—N1	2.127 (4)	C18—C23	1.397 (9)
Fe1—N4	2.161 (4)	C19—C20	1.381 (10)
Cl1—C4	1.729 (6)	C19—H19	0.930
Cl2—C27	1.743 (6)	C20—C21	1.408 (10)
Na1—O5^i^	2.222 (4)	C20—H20	0.930
Na1—O6	2.238 (5)	C21—C22	1.373 (11)
Na1—O7	2.508 (7)	C21—H21	0.930
Na1—O8	2.329 (6)	C22—C23	1.404 (9)
Na1—N7	2.381 (9)	C22—H22	0.930
O1—C7	1.304 (6)	C23—H23	0.930
O2—C24	1.320 (6)	C24—C25	1.407 (8)
O3—C30	1.312 (6)	C24—C29	1.424 (7)
O4—C1	1.344 (7)	C25—C26	1.375 (8)
O5—C40	1.253 (7)	C25—H25	0.930
O6—C17	1.258 (8)	C26—C27	1.413 (8)
N1—N2	1.290 (6)	C26—H26	0.930
N1—C6	1.420 (9)	C27—C28	1.375 (8)
N2—C8	1.384 (8)	C28—C29	1.398 (8)
N3—C17	1.361 (7)	C28—H28	0.930
N3—C18	1.424 (8)	C30—C31	1.406 (8)
N3—H3N	0.860	C30—C39	1.460 (8)
N4—N5	1.289 (5)	C31—C32	1.455 (7)
N4—C29	1.409 (7)	C32—C33	1.412 (8)
N5—C31	1.380 (7)	C32—C37	1.418 (7)
N6—C40	1.353 (7)	C33—C34	1.373 (7)
N6—C41	1.407 (7)	C33—H33	0.930
N6—H6N	0.860	C34—C35	1.406 (8)
N7—C47	1.130 (13)	C34—H34	0.930
C1—C2	1.404 (10)	C35—C36	1.363 (9)
C1—C6	1.400 (8)	C35—H35	0.930
C2—C3	1.382 (8)	C36—C37	1.408 (7)
C2—H2	0.930	C36—H36	0.930
C3—C4	1.401 (8)	C37—C38	1.408 (8)
C3—H3	0.930	C38—C39	1.366 (7)
C4—C5	1.376 (10)	C38—H38	0.930
C5—C6	1.404 (8)	C39—C40	1.511 (9)
C5—H5	0.930	C41—C42	1.390 (8)
C7—C8	1.420 (8)	C41—C46	1.379 (7)
C7—C16	1.449 (9)	C42—C43	1.387 (9)
C8—C9	1.441 (7)	C42—H42	0.930
C9—C10	1.417 (8)	C43—C44	1.393 (8)
C9—C14	1.410 (10)	C43—H43	0.930
C10—C11	1.379 (9)	C44—C45	1.382 (8)
C10—H10	0.930	C44—H44	0.930
C11—C12	1.380 (11)	C45—C46	1.379 (9)
C11—H11	0.930	C45—H45	0.930
C12—C13	1.374 (10)	C46—H46	0.930
C12—H12	0.930	C47—C48	1.472 (14)
C13—C14	1.424 (8)	C48—H48A	0.960
C13—H13	0.930	C48—H48B	0.960
C14—C15	1.417 (9)	C48—H48C	0.960
			
O4···O9^ii^	2.710 (6)	O9···O4^iii^	2.710 (6)
O8···O9	2.730 (7)	O9···O8	2.730 (7)
			
O1—Fe1—O2	90.26 (17)	N3—C17—C16	117.7 (6)
O1—Fe1—O3	85.60 (17)	N3—C18—C19	124.4 (5)
O1—Fe1—O4	160.14 (15)	N3—C18—C23	116.0 (6)
O1—Fe1—N1	81.72 (18)	C19—C18—C23	119.5 (5)
O1—Fe1—N4	111.90 (19)	C18—C19—C20	120.8 (6)
O2—Fe1—O3	155.71 (17)	C18—C19—H19	119.9
O2—Fe1—O4	96.86 (18)	C20—C19—H19	119.3
O2—Fe1—N1	100.35 (16)	C19—C20—C21	119.9 (7)
O2—Fe1—N4	79.14 (16)	C19—C20—H20	120.1
O3—Fe1—O4	95.02 (17)	C21—C20—H20	120.1
O3—Fe1—N1	102.70 (16)	C20—C21—C22	119.5 (6)
O3—Fe1—N4	80.26 (17)	C20—C21—H21	120.2
O4—Fe1—N1	78.78 (18)	C22—C21—H21	120.2
O4—Fe1—N4	87.69 (18)	C21—C22—C23	120.7 (6)
N1—Fe1—N4	166.3 (2)	C21—C22—H22	119.6
O5^i^—Na1—O6	97.7 (2)	C23—C22—H22	119.6
O5^i^—Na1—O7	88.35 (19)	C18—C23—C22	119.5 (7)
O5^i^—Na1—O8	108.0 (2)	C18—C23—H23	120.3
O5^i^—Na1—N7	149.5 (3)	C22—C23—H23	120.3
O6—Na1—O7	92.9 (2)	O2—C24—C25	123.0 (4)
O6—Na1—O8	101.3 (2)	O2—C24—C29	119.4 (4)
O6—Na1—N7	107.6 (2)	C25—C24—C29	117.6 (5)
O7—Na1—O8	156.4 (2)	C24—C25—C26	120.8 (5)
O7—Na1—N7	73.9 (2)	C24—C25—H25	119.6
O8—Na1—N7	83.8 (2)	C26—C25—H25	119.6
Fe1—O1—C7	136.1 (4)	C25—C26—C27	120.2 (5)
Fe1—O2—C24	117.8 (3)	C25—C26—H26	119.9
Fe1—O3—C30	130.3 (4)	C27—C26—H26	119.9
Fe1—O4—C1	116.7 (3)	Cl2—C27—C26	118.6 (4)
Na1^i^—O5—C40	159.8 (5)	Cl2—C27—C28	120.6 (4)
Na1—O6—C17	145.0 (3)	C26—C27—C28	120.8 (5)
Fe1—N1—N2	133.6 (4)	C27—C28—C29	118.8 (5)
Fe1—N1—C6	112.0 (3)	C27—C28—H28	120.6
N2—N1—C6	114.3 (4)	C29—C28—H28	120.6
N1—N2—C8	120.5 (4)	N4—C29—C24	112.6 (4)
C17—N3—C18	129.2 (6)	N4—C29—C28	125.6 (5)
C17—N3—H3N	115.4	C24—C29—C28	121.7 (5)
C18—N3—H3N	115.4	O3—C30—C31	123.1 (5)
Fe1—N4—N5	132.3 (3)	O3—C30—C39	118.8 (5)
Fe1—N4—C29	110.8 (3)	C31—C30—C39	118.1 (4)
N5—N4—C29	115.6 (4)	N5—C31—C30	125.8 (4)
N4—N5—C31	119.2 (4)	N5—C31—C32	113.0 (5)
C40—N6—C41	129.3 (4)	C30—C31—C32	121.1 (5)
C40—N6—H6N	115.3	C31—C32—C33	123.2 (5)
C41—N6—H6N	115.4	C31—C32—C37	118.6 (5)
Na1—N7—C47	171.9 (8)	C33—C32—C37	118.2 (4)
O4—C1—C2	122.4 (5)	C32—C33—C34	120.4 (5)
O4—C1—C6	119.0 (6)	C32—C33—H33	119.8
C2—C1—C6	118.5 (5)	C34—C33—H33	119.8
C1—C2—C3	119.7 (5)	C33—C34—C35	121.9 (5)
C1—C2—H2	120.1	C33—C34—H34	119.0
C3—C2—H2	120.1	C35—C34—H34	119.0
C2—C3—C4	120.8 (6)	C34—C35—C36	117.9 (5)
C2—C3—H3	119.6	C34—C35—H35	121.1
C4—C3—H3	119.6	C36—C35—H35	121.1
Cl1—C4—C3	119.3 (5)	C35—C36—C37	122.6 (5)
Cl1—C4—C5	120.0 (4)	C35—C36—H36	118.7
C3—C4—C5	120.7 (5)	C37—C36—H36	118.7
C4—C5—C6	118.3 (5)	C32—C37—C36	119.0 (5)
C4—C5—H5	120.8	C32—C37—C38	119.2 (4)
C6—C5—H5	120.8	C36—C37—C38	121.8 (5)
N1—C6—C1	112.8 (5)	C37—C38—C39	123.0 (5)
N1—C6—C5	125.3 (5)	C37—C38—H38	118.5
C1—C6—C5	121.9 (6)	C39—C38—H38	118.5
O1—C7—C8	121.8 (6)	C30—C39—C38	119.8 (5)
O1—C7—C16	119.5 (5)	C30—C39—C40	123.5 (4)
C8—C7—C16	118.7 (5)	C38—C39—C40	116.7 (5)
N2—C8—C7	125.9 (5)	O5—C40—N6	123.1 (5)
N2—C8—C9	113.4 (5)	O5—C40—C39	119.5 (5)
C7—C8—C9	120.8 (6)	N6—C40—C39	117.3 (4)
C8—C9—C10	122.4 (6)	N6—C41—C42	124.1 (4)
C8—C9—C14	118.9 (5)	N6—C41—C46	117.0 (4)
C10—C9—C14	118.7 (5)	C42—C41—C46	118.9 (5)
C9—C10—C11	119.3 (6)	C41—C42—C43	119.3 (5)
C9—C10—H10	120.4	C41—C42—H42	120.3
C11—C10—H10	120.4	C43—C42—H42	120.3
C10—C11—C12	122.8 (6)	C42—C43—C44	121.6 (6)
C10—C11—H11	118.6	C42—C43—H43	119.2
C12—C11—H11	118.6	C44—C43—H43	119.2
C11—C12—C13	119.0 (6)	C43—C44—C45	118.3 (6)
C11—C12—H12	120.5	C43—C44—H44	120.8
C13—C12—H12	120.5	C45—C44—H44	120.8
C12—C13—C14	120.7 (7)	C44—C45—C46	120.1 (5)
C12—C13—H13	119.5	C44—C45—H45	119.9
C14—C13—H13	119.8	C46—C45—H45	120.0
C9—C14—C13	119.6 (5)	C41—C46—C45	121.8 (5)
C9—C14—C15	119.3 (5)	C41—C46—H46	119.1
C13—C14—C15	121.1 (6)	C45—C46—H46	119.1
C14—C15—C16	123.0 (6)	N7—C47—C48	179.8 (9)
C14—C15—H15	118.7	C47—C48—H48A	109.5
C16—C15—H15	118.3	C47—C48—H48B	109.5
C7—C16—C15	119.3 (5)	C47—C48—H48C	109.5
C7—C16—C17	124.6 (5)	H48A—C48—H48B	109.5
C15—C16—C17	116.1 (6)	H48A—C48—H48C	109.5
O6—C17—N3	121.1 (5)	H48B—C48—H48C	109.5
O6—C17—C16	121.2 (5)		
			
O1—Fe1—O2—C24	−116.4 (4)	C3—C4—C5—C6	2.7 (8)
O2—Fe1—O1—C7	−107.1 (5)	C4—C5—C6—N1	179.9 (4)
O1—Fe1—O3—C30	145.6 (5)	C4—C5—C6—C1	−1.8 (8)
O3—Fe1—O1—C7	96.8 (5)	O1—C7—C8—N2	0.3 (6)
O1—Fe1—O4—C1	−18.3 (7)	O1—C7—C8—C9	−179.5 (4)
O4—Fe1—O1—C7	4.2 (8)	O1—C7—C16—C15	−179.8 (3)
O1—Fe1—N1—N2	4.6 (4)	O1—C7—C16—C17	2.3 (8)
O1—Fe1—N1—C6	−178.2 (3)	C8—C7—C16—C15	1.8 (7)
N1—Fe1—O1—C7	−6.7 (5)	C8—C7—C16—C17	−176.1 (5)
O1—Fe1—N4—N5	−105.7 (6)	C16—C7—C8—N2	178.7 (4)
O1—Fe1—N4—C29	88.5 (4)	C16—C7—C8—C9	−1.2 (7)
N4—Fe1—O1—C7	174.5 (4)	N2—C8—C9—C10	−2.1 (7)
O2—Fe1—O3—C30	64.8 (7)	N2—C8—C9—C14	−179.5 (4)
O3—Fe1—O2—C24	−36.6 (7)	C7—C8—C9—C10	177.8 (5)
O2—Fe1—O4—C1	91.9 (3)	C7—C8—C9—C14	0.4 (6)
O4—Fe1—O2—C24	82.2 (4)	C8—C9—C10—C11	−178.4 (5)
O2—Fe1—N1—N2	93.4 (4)	C8—C9—C14—C13	178.9 (5)
O2—Fe1—N1—C6	−89.4 (3)	C8—C9—C14—C15	−0.2 (6)
N1—Fe1—O2—C24	162.0 (4)	C10—C9—C14—C13	1.4 (8)
O2—Fe1—N4—N5	168.5 (6)	C10—C9—C14—C15	−177.7 (5)
O2—Fe1—N4—C29	2.6 (4)	C14—C9—C10—C11	−1.0 (8)
N4—Fe1—O2—C24	−4.1 (4)	C9—C10—C11—C12	0.2 (7)
O3—Fe1—O4—C1	−109.3 (3)	C10—C11—C12—C13	0.2 (7)
O4—Fe1—O3—C30	−54.3 (5)	C11—C12—C13—C14	0.2 (7)
O3—Fe1—N1—N2	−78.9 (4)	C12—C13—C14—C9	−1.0 (9)
O3—Fe1—N1—C6	98.3 (3)	C12—C13—C14—C15	178.1 (6)
N1—Fe1—O3—C30	−133.9 (5)	C9—C14—C15—C16	0.9 (8)
O3—Fe1—N4—N5	−24.5 (6)	C13—C14—C15—C16	−178.2 (5)
O3—Fe1—N4—C29	169.7 (4)	C14—C15—C16—C7	−1.7 (8)
N4—Fe1—O3—C30	32.5 (5)	C14—C15—C16—C17	176.4 (5)
O4—Fe1—N1—N2	−171.6 (4)	C7—C16—C17—O6	169.3 (5)
O4—Fe1—N1—C6	5.6 (3)	C7—C16—C17—N3	−9.5 (7)
N1—Fe1—O4—C1	−7.3 (3)	C15—C16—C17—O6	−8.7 (7)
O4—Fe1—N4—N5	71.0 (6)	C15—C16—C17—N3	172.5 (4)
O4—Fe1—N4—C29	−94.8 (4)	N3—C18—C19—C20	179.7 (5)
N4—Fe1—O4—C1	170.7 (3)	N3—C18—C23—C22	178.8 (5)
N1—Fe1—N4—N5	79.3 (10)	C19—C18—C23—C22	−1.7 (8)
N1—Fe1—N4—C29	−86.5 (7)	C23—C18—C19—C20	0.2 (7)
N4—Fe1—N1—N2	179.9 (5)	C18—C19—C20—C21	1.1 (9)
N4—Fe1—N1—C6	−2.9 (9)	C19—C20—C21—C22	−1.0 (9)
O5^i^—Na1—O6—C17	−15.9 (6)	C20—C21—C22—C23	−0.4 (8)
O6—Na1—O5^i^—C40^i^	−161.4 (11)	C21—C22—C23—C18	1.8 (8)
O7—Na1—O5^i^—C40^i^	−68.7 (12)	O2—C24—C25—C26	178.6 (6)
O8—Na1—O5^i^—C40^i^	94.0 (12)	O2—C24—C29—N4	−2.4 (9)
N7—Na1—O5^i^—C40^i^	−15.1 (14)	O2—C24—C29—C28	−179.6 (6)
O7—Na1—O6—C17	−104.7 (6)	C25—C24—C29—N4	177.1 (6)
O8—Na1—O6—C17	94.3 (6)	C25—C24—C29—C28	−0.0 (9)
N7—Na1—O6—C17	−178.7 (6)	C29—C24—C25—C26	−1.0 (10)
Fe1—O1—C7—C8	5.8 (8)	C24—C25—C26—C27	1.3 (10)
Fe1—O1—C7—C16	−172.5 (3)	C25—C26—C27—Cl2	179.2 (5)
Fe1—O2—C24—C25	−174.4 (5)	C25—C26—C27—C28	−0.7 (10)
Fe1—O2—C24—C29	5.1 (8)	Cl2—C27—C28—C29	179.9 (4)
Fe1—O3—C30—C31	−29.3 (10)	C26—C27—C28—C29	−0.3 (8)
Fe1—O3—C30—C39	151.0 (5)	C27—C28—C29—N4	−176.1 (6)
Fe1—O4—C1—C2	−173.9 (4)	C27—C28—C29—C24	0.6 (10)
Fe1—O4—C1—C6	8.1 (6)	O3—C30—C31—N5	0.9 (12)
Na1^i^—O5—C40—N6	−117.5 (11)	O3—C30—C31—C32	−179.8 (6)
Na1^i^—O5—C40—C39	65.3 (14)	O3—C30—C39—C38	−176.9 (6)
Na1—O6—C17—N3	128.1 (5)	O3—C30—C39—C40	2.4 (10)
Na1—O6—C17—C16	−50.6 (8)	C31—C30—C39—C38	3.4 (10)
Fe1—N1—N2—C8	−1.7 (7)	C31—C30—C39—C40	−177.3 (7)
Fe1—N1—C6—C1	−3.2 (5)	C39—C30—C31—N5	−179.4 (6)
Fe1—N1—C6—C5	175.3 (4)	C39—C30—C31—C32	−0.0 (9)
N2—N1—C6—C1	174.6 (4)	N5—C31—C32—C33	−2.4 (10)
N2—N1—C6—C5	−7.0 (7)	N5—C31—C32—C37	176.3 (6)
C6—N1—N2—C8	−178.9 (4)	C30—C31—C32—C33	178.2 (7)
N1—N2—C8—C7	−2.0 (7)	C30—C31—C32—C37	−3.1 (10)
N1—N2—C8—C9	177.9 (4)	C31—C32—C33—C34	177.1 (7)
C17—N3—C18—C19	13.2 (8)	C31—C32—C37—C36	−177.0 (6)
C17—N3—C18—C23	−167.3 (5)	C31—C32—C37—C38	2.9 (10)
C18—N3—C17—O6	−2.3 (8)	C33—C32—C37—C36	1.7 (10)
C18—N3—C17—C16	176.5 (4)	C33—C32—C37—C38	−178.3 (7)
Fe1—N4—N5—C31	10.9 (9)	C37—C32—C33—C34	−1.6 (11)
Fe1—N4—C29—C24	−0.9 (7)	C32—C33—C34—C35	−0.3 (9)
Fe1—N4—C29—C28	176.0 (5)	C33—C34—C35—C36	1.9 (12)
N5—N4—C29—C24	−169.4 (5)	C34—C35—C36—C37	−1.7 (12)
N5—N4—C29—C28	7.6 (10)	C35—C36—C37—C32	−0.0 (10)
C29—N4—N5—C31	176.2 (6)	C35—C36—C37—C38	180.0 (7)
N4—N5—C31—C30	7.2 (11)	C32—C37—C38—C39	0.4 (11)
N4—N5—C31—C32	−172.2 (6)	C36—C37—C38—C39	−179.6 (7)
C40—N6—C41—C42	−2.0 (12)	C37—C38—C39—C30	−3.7 (11)
C40—N6—C41—C46	177.4 (7)	C37—C38—C39—C40	177.0 (7)
C41—N6—C40—O5	−2.7 (12)	C30—C39—C40—O5	−165.1 (7)
C41—N6—C40—C39	174.5 (6)	C30—C39—C40—N6	17.5 (11)
O4—C1—C2—C3	−178.3 (5)	C38—C39—C40—O5	14.1 (11)
O4—C1—C6—N1	−2.8 (7)	C38—C39—C40—N6	−163.2 (7)
O4—C1—C6—C5	178.7 (5)	N6—C41—C42—C43	179.6 (7)
C2—C1—C6—N1	179.0 (4)	N6—C41—C46—C45	−179.3 (6)
C2—C1—C6—C5	0.5 (8)	C42—C41—C46—C45	0.1 (8)
C6—C1—C2—C3	−0.2 (6)	C46—C41—C42—C43	0.2 (8)
C1—C2—C3—C4	1.1 (8)	C41—C42—C43—C44	−0.4 (10)
C2—C3—C4—Cl1	177.8 (4)	C42—C43—C44—C45	0.3 (9)
C2—C3—C4—C5	−2.4 (8)	C43—C44—C45—C46	0.1 (10)
Cl1—C4—C5—C6	−177.5 (4)	C44—C45—C46—C41	−0.3 (9)

Symmetry codes: (i) −*x*+1, −*y*, −*z*+1; (ii) *x*, *y*+1, *z*; (iii) *x*, *y*−1, *z*.

### Hydrogen-bond geometry (Å, °)

**Table:**

*D*—H···*A*	*D*—H	H···*A*	*D*···*A*	*D*—H···*A*
N3—H3N···O1	0.86	1.94	2.644 (7)	138
N6—H6N···O3	0.86	1.97	2.664 (6)	138
O8—H···O9	.	.	2.730 (7)	.
O9—H···O4^iii^	.	.	2.710 (6)	.

Symmetry codes: (iii) *x*, *y*−1, *z*.
