# Diacetonitrile­tetra­kis{μ_2_-3-anilinocarbonyl-1-[(5-chloro-2-oxidophen­yl)diazen­yl]-2-naphtholato}tetra­aqua­diiron(III)disodium(I) dihydrate. Corrigendum

**DOI:** 10.1107/S1600536808002869

**Published:** 2008-01-30

**Authors:** Yohei Sato, Kazuya Uta, Jin Mizuguchi

**Affiliations:** a Department of Applied Physics, Graduate School of Engineering, Yokohama National University, 79-5 Tokiwadai, Hodogaya-ku, 240-8501 Yokohama, Japan

## Abstract

Corrections are made to the names of the first two authors in Yohei, Kazuya & Mizuguchi [*Acta Cryst.* (2008), E**64**, m240–m241].

## Abstract 2

Corrigendum to *Acta Cryst.* (2008), E**64**, m240–m241.

In the paper by Yohei, Kazuya & Mizuguchi [*Acta Cryst.* (2008), E**64**, m240–m241], the names of the first two authors are given incorrectly. The correct names should be Yohei Sato and Kazuya Uta, as given above.

